# Exploration of photoprotective and antibiotic activity of wild Polypodiaceae ferns from Costa Rica

**DOI:** 10.1038/s41598-023-50281-3

**Published:** 2024-01-18

**Authors:** Yaclyn Salazar-Chacón, Maria José Gutierrez-Bolaños, Jimena Padilla-Cordero, Camilo Vidaurre-Rodriguez, Yendry Carvajal-Miranda, Alexander Rojas-Alvarado, Jorengeth Abad Rodríguez-Rodríguez, Gerardo Rodríguez-Rodríguez, Víctor Álvarez-Valverde, Pablo Jiménez-Bonilla

**Affiliations:** 1 Laboratorio de Fitoquímica (LAFIT), Escuela de Química, Universidad Nacional (UNA), Campus Omar Dengo, Heredia, 83-3000 Costa Rica; 2 Agricultural Chemistry Department, University of California Davis, Davis, CA USA; 3 Universidad Nacional de Costa Rica, Campus Omar Dengo, Heredia, 83-3000 Costa Rica; 4 Laboratorio de Biotecnología Microbiana (LABIMI), Escuela de Ciencias Biológicas, Universidad Nacional (UNA), Campus Omar Dengo, Heredia, 83-3000 Costa Rica; 5 Laboratorio de Productos Naturales y Ensayos Biológicos (LAPRONEB), Escuela de Química, Universidad Nacional (UNA), Campus Omar Dengo, Heredia, 83-3000 Costa Rica; 6 Escuela de Química, Universidad Nacional (UNA), New Industrial Processes Bldg, Omar Dengo Campus, Heredia, 40101 Costa Rica

**Keywords:** Medicinal chemistry, Natural products, Plant sciences

## Abstract

Skin disorders affect millions of people all over the world. There are limited options to treat dermal illnesses such as vitiligo, psoriasis, and atopic dermatitis (eczema). Central American ferns are a potential source of bioactive metabolites against those diseases. Currently, *Polypodium leucotomos* Poir. is the only one being commercially utilized for this purpose. In this work, we evaluated the concentration of the skin bioactive compounds: quinic and chlorogenic acid, in the extract of 20 wild ferns from Costa Rica. We also evaluated the antimicrobial capabilities of the crude extracts of wild ferns and the sun protection factor (SPF) of the extracts. We found 19 out of 20 have either an important concentration of the compounds mentioned above or antimicrobial properties. Also, most samples result in higher SPF than *P. aureum*’s rhizome. We also have studied the fern acclimatization, at different shading conditions, finding a significant influence of the culturing conditions on metabolite production. After acclimatization. So far, we demonstrate that various ferns included in this study are a potential source of treatments for skin conditions.

## Introduction

Ferns are primitive non-flowering vascular plants, and many of them have shown medicinal properties. Several ferns have been studied as bio-herbicides, hepatoprotectives, cytotoxic, antihyperglycemic, trypanocidal, antimicrobial, antinociceptive, and immunomodulatory^1^. Nutritional, nutraceutical and pharmacological utilization are still limited at a commercial scale, with some exceptions, such as kalawalla (*Phlebodium aureum* formerly known as *Polypodium leucotomos*). The extract of *P. aureum* has been used to treat skin diseases since the ‘80s, and there are many commercial preparations based on the hydrosoluble extract of their rhizomes, such as Fernblock®, Heliocare®, Difur®, and others. *P. aureum* extracts have been commercialized mainly in Europe despite this plant is endemic to Central America^2^.

Oral or topic formulations containing *P. aureum* extract are recognized for their photoprotectant bioactivity and their strong antioxidant properties, which have been related to their naturally occurring phenolic compounds such as *p*-coumaric, ferulic, caffeic, vanillic, and chlorogenic acid^3^. Commercial aqueous extract of the leaves of *P. aureum (*marketed as Fernblock®, Fernplus®, Fernmed®, and Fernage®) contain a standardized 0.6–1.3% total phenolic compounds concentration and 0.4–0.9% quinic acid concentration^4^. Mechanisms of action of these commercial products involve free radical scavenging and regulation of several genes, such as metalloproteinases^5^, tyrosinase inhibition^6^, and others. Also, natural phenolic compounds are known for absorbing UV radiation, similar to synthetic compounds, such as homosalate or dibenzalacetone do so. Then fern extracts are an excellent alternative for bio-based wide-spectrum sun blockers and sunscreen^7^. Also, fern extracts have been found active to prevent UV damage during clinical trials^8^, preventing sunburns, and subsequently skin cancer.

Fern-derived nutraceutical products are an alternative for some skin disorders with unavailable treatment options, such as eczema or vitiligo. Also, skin microbiome imbalance and traditional antibiotic utilization contribute to developing and speeding up skin depigmentation^9^. For example, *Staphylococcus* spp*.* is more abundant on the skin of patients with vitiligo^10,11^. Some aqueous fern rhizome extracts showed antibacterial properties against gram-negative bacteria (such as *Escherichia coli*) and gram-positive (such as *S. aureus*)^12^.

Other Central American Polypodiaceae and Pteridophyta ferns have similar metabolic pathways to *P. aureum*^13^, and also have been used for the same applications in traditional medicine^14^. Although some wild ferns are not feasible to reproduce in a greenhouse, there is an enormous potential for the discovery and commercialization of medicinal ferns in Central America^15^. Also, growing conditions such as climate, altitude, light incidence, and substrate composition can be used to improve or standardize metabolite production^16,17^. Therefore, we hypothesize that other ferns can have similar or superior activity compared to *P. aureum*, in terms of UV protection and metabolite concentration. Some of them can be potentially more active or more economic to produce than kalawalla is. In this work, we explored wild and acclimatized ferns in terms of amounts of quinic acid, chlorogenic acid, and total polyphenolic compounds, as potential alternatives to treat skin disorders.

## Materials and methods

### Microorganism strains

The strains utilized in this study were *Staphylococcus aureus* ATCC 25923, *Staphylococcus epidermidis* ATCC 12228, *Pseudomonas aeruginosa* ATCC 9027, and *Escherichia coli* ATCC 25922.

### Chemicals and materials

Quinic acid, chlorogenic acid, Folin-Ciocalteu’s reagent, and gallic acid were obtained from Sigma-Aldrich (MS, USA). Methanol and acetone were purchased from JT Baker (PA, USA). Solution 1000 μg/mL of Penicillin/Streptomycin were acquired from GIBCO (NY, USA), Mueller Hinton Agar was purchased from OXOID LTD (Hampshire, Ireland). Nivea Sun®, Eucerin®, and Vicky face® sunscreens were manufactured by Beiersdorf UK Ltd (Birhmingham, UK), Beiersdorf Int. (Hamburg, Germany), and Vickystore (NJ, USA), respectively.

### Fern samples

**Figure 1:**
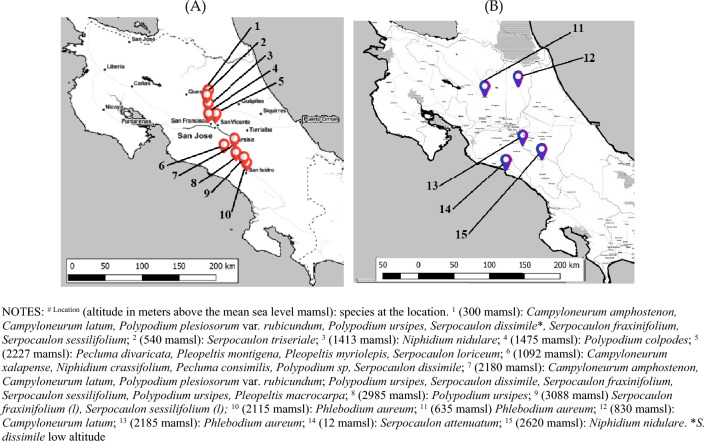
Sampling locations for biological material. (**A**) Wild ferns. (**B**) Acclimatized ferns. Maps constructed using Leaflet Map tiles by Stamen Design, CC BY 3.0 OpenStreetMap Contributors.

Twenty wild native fern species were collected from the forest at 13 locations. Figure 1A summarizes the identified species and their location. Both rhizomes and leaves were collected for each plant, oven-dried for 72 h at 40 °C, and ground to 1 mm.

### Quinic and chlorogenic acid determination

0.5 g dry material (rhizome or leaves) was extracted in 1.5 mL 75% methanol. Extraction protocol has been optimized as described in Supplementary Materials and Fig. S3. Afterwards, it was filtered through a 0.2 µm membrane. The extract was dried into an analytic evaporator ZipVap Zanntek (from Glas-Col®, Terre Haute, IN) at 40 °C under nitrogen. Then, it was reconstituted in 500 µL 75% methanol. The reconstituted extract was analyzed by HPLC, using a solvent gradient described in Table 1. The column utilized is an Acclaim 120 (C-18 (4.6 × 150) mm, 5 μm) (from Thermo Scientific Dionex, CA) at 35 °C. A diode array detector was used to detect quinic acid (QA) and chlorogenic acid (CGA) at 215 and 325 nm, respectively, against 0–2000 mg/L QA and 0–600 mg/L CGA standard curves.

**Table 1:** Starting, interception and ending points for linear gradients of mobile phase, utilized to quantify quinic and chlorogenic acid through HPLC.

Point	Time (min)	Flow rate	Acetonitrile (%)	Metanol (%)	0.1%TFA (%)
Starting	0	0.8	10	0	90
Intersection	5	0.8	10	10	80
Intersection	8	0.8	10	20	70
Intersection	10	0.8	10	40	50
Intersection	13.5	1.5	10	10	80
Ending	15	0.8	10	0	90

### Total polyphenolic content (TPC) quantification

0.1 g of each sample was mixed with 3 mL of 75% methanol and sonicated for 10 min. Then, the mixture was centrifuged for 5 min, and the methanolic extract was collected. The remaining solid was extracted two additional times following the same procedure. All the extracts were combined and diluted to complete 10 mL (total volume). Then, Folin–Ciocalteu’s colorimetric method was used to determine total phenolic content, as described in our previous work^18^, using gallic acid as standard. Each sample has three replicates. Both *S. dissimile* specimens were combined in equal amounts for this analysis.

### Antibiotic activity test

In two replicates, we followed the agar well diffusion method^19^, a modification of the Kirby-Bauer susceptibility protocol^20^. These experiments were conducted on Mueller Hinton agar plates. 100 µL of each microorganism at 0.5 of McFarlan scale (1.5 × 10^8^ UFC/mL) in NaCl 0.85% were plated into a sterile 90 × 15 mm petri dish containing 25 mL of Mueller Hinton agar. Then, four 6 mm wells were made in the agar at the same distance, three wells were filled with 50 μL of 0.1 g/mL fern extract, and the fourth well was filled with 50 μL of 50%v/v acetone (as negative control). Additionally, for the positive control, the same procedure was carried out, but 3 well were filled with 50 μL of 1000 μg/mL of Penicillin/Streptomycin, and the fourth well was filled with 50 μL of 50%v/v acetone (as negative control). Plates were incubated for 24 h at 35 °C, and inhibition rings were measured. Two replicates were used for each condition. Microbial activity was calculated according to (1).

1 $$\mathrm{\%}RPI=\frac{({D}_{sample}-{D}_{negative\, control})}{({D}_{positive \,control})}\times 100.$$

%*RPI* is the Relative Percentage of Inhibition, and *D*_*sample*_, *D*_*negative control*_, and *D*_*positive control*_ are the diameters of the inhibition zones indicated in the subscripts.

### Solar protection factor (SPF) determination

The same extracts prepared for TPC analysis were diluted 100 times, and the UV–Vis spectra were recorded for each sample repetition between 190 and 800 nm in a spectrophotometer Evolution 350 (from Thermo Scientific, Massachusetts, USA). Data were exported and analyzed according to Mansur equation^21^. SPFs were adjusted to the corresponding concentration found in the dry material (leaves or rhizome), and the average of 3 commercial sunscreens (SPF 50) was used as reference: Nivea Sun®, Eucerin®, and Vicky face® sunscreens. Three repetitions had been used.

### Acclimatization of selected ferns

Six fern species were selected to grow under controlled conditions to study the acclimatization effect over metabolite production. *Campyloneurum latum* T. Moore, *Niphidium nidulare* (Rosenst.) Lellinger, *Phlebodium pseudoaureum* (Cav.) Lellinger, *Serpocaulon attenuatum* (C. Presl) A.R. Sm., *S. sessilifolium* (Desv.) A.R. Sm. y *S. triseriale* (Sw.) A.R. Sm. were collected from the wild at the locations mentioned in Fig. 1B. The samples were reproduced at Macho River Biological Station, located at 9° 46ʹ 00ʹʹ N, 83° 46ʹ 00ʹʹ W, a 1600 mamsl, in Orosí, Cartago, Costa Rica. The reported temperature range at the location is 18.3–26.5 °C (average 22.3 °C), and the annual precipitation is 3120 mm. 68 plants of each specie were distributed under 50% and 70% shade (covered with saran fabric) in 1–3 L plant pots containing a substrate composed by: peat (1/4), organic fertilizer (1/4), grounded coconut fiber (1/4) and coconut shell chunks (1/4). They were harvested after a year, processed, and analyzed using the same methods described for wild ferns.

### Statistical analysis

All assays were evaluated using three replicates. Results of TPC, quinic acid, chlorogenic acid, SPF, and antibiotic activities are reported as average ± standard deviation. R statistical software were used to calculate Tuckey’s post hoc test. Compact letter display is used to express Tukey’s results. Samples with same letter does not contain significant differences, using α = 0.05.

### Ethical declarations

The plants utilized in field experiments accomplish the institutional and national legislation. The protected vegetal material is authorized by the National Committee for the Management of Biodiversity (CONAGEBIO) of Costa Rica through the resolution R-CM-UNA-004-2018-OT.

## Results and discussion

### Content of quinic and chlorogenic acids of wild ferns

**Figure 2:**
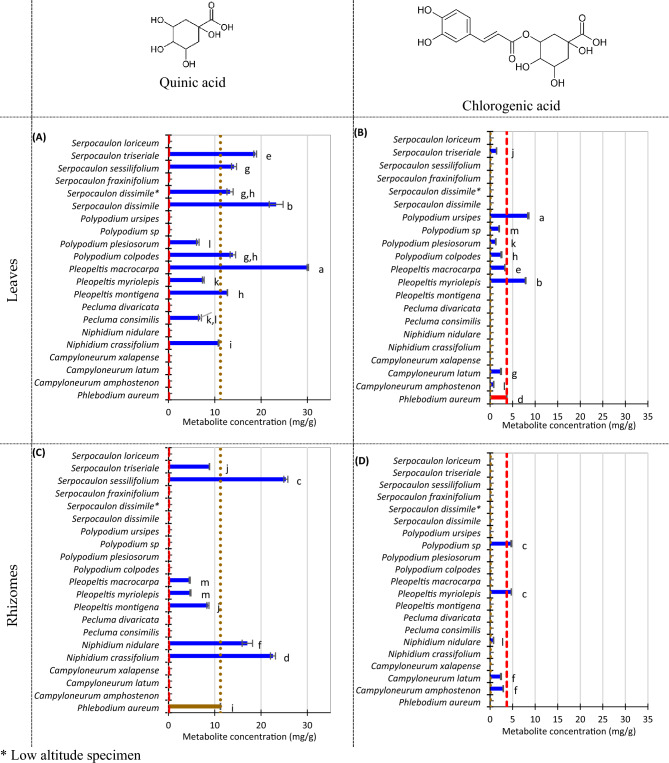
Selected metabolite concentration of Costa Rican wild ferns samples (dry base). (**A**) Quinic acid content of fern leaves. (**B**) Chlorogenic acid content of fern leaves. (**C**) Quinic acid content of fern rhizomes. (**D**) Chlorogenic acid content of fern rhizomes. Error bars represent standard deviation. Dashed and dotted lines represent *Phlebodium aureum* leaves and rhizome concentration, respectively. Compact letter display beside the column represents Tuckey’s test divided into quinic acid and chlorogenic acid. α = 0.05.

Figure 2 summarizes the metabolite content of leaves and rhizome of the 20 wild ferns included in this study. *Polypodium leucotomos* is a synonym for *Phlebodium pseudoaureum* (sensu stricto) or *Phlebodium aureum* (sensu lato).

Quinic acid was present in 11 leaf extracts and eight rhizome extracts. Notwithstanding the commercial product elaborated from the rhizome extract, we decided to include the leaves, considering that metabolites could be distributed all over the ferns. Interestingly, quinic acid was detected in leaves of *Pecluma consimilis* (Mett.) M.G. Price, *Polypodium colpodes* Kunze, *P. plesiosorum* Kunze and *Serpocaulon dissimile* (L.) A.R. Sm., but it was not detected in the rhizomes of those species. Also, quinic acid was detected in both rhizomes and leaves of *Niphidium crassifolium* (L.) Lellinger, *Pleopeltis macrocarpa* (Bory ex Willd.) Kaulf., *P. montingena* (Maxon) A.R. Sm. & Tejero, *P. myriolepis* (Christ) A.R. Sm. & Tejero*, Serpocaulon sessilifolium* (Desv.) A.R. Sm. and *S. triseriale* (Sw.) A.R. Sm., but the concentration of leaves was higher in most of them, except for *N. crassifolium* and *S. sessilifolium*.

Quinic acid was not detected in *P. aureum* leaves, but it accounts for 11.2 mg/g in the rhizome. Figure 2A contains the quinic acid concentration found on fern leaves. Three species showed a quinic acid content 56–67% lower, respecting our reference value (*P. aureum* rhizome): *P. consimilis*, *P. myriolepis,* and *P. plesiosorum*. Another five species have ranged from similar levels to 30% higher quinic acid content (than *P. aureum* rhizome): *N. crassifolium*, *P. montigena*, *P. colpodes*, *S. dissimile*, and *S. sessilifolium*. Finally, the leaves of three fern species contain between 1.7 to 2.7-fold-up respecting *P. aureum* rhizome: *P. macrocarpa*, *S. dissimile,* and *S. triseriale*.

Figure 2C shows quinic acid in rhizomes. Four species (*P. macrocarpa*, *P. montigena P. myriolepis*, and *S. triseriale*) contain lower quinic acid than *P. aureum*. But also, three species contain 1.5–2.3 higher quinic acid than *P. aureum*: *N. crassifolium*, *N. nidulare,* and *S. sessilifolium.* According to the literature, an analysis of Fernblock®, the commercial extract from *P. aureum* accounts for 70 g/L quinic acid. The three species with higher quinic acid concentration are potentially a more economical source of this metabolite^4^.

Also, phenolic compounds account for 1% of commercial Fernblock®^4^, composed mainly of 3,4-dihydroxybenzoic acid, 4-hydroxybenzoic acid, vanillic acid, caffeic acid, *p-*coumaric acid, ferulic acid, 4-hydroxycinnamoyl-quinic acid, and five isomers of chlorogenic acid^22^. Chlorogenic acid is a dietary polyphenol^23^, linked with activity against inflammation, skin diseases, and others^23,24^, so we had included its quantification in this study. Chlorogenic acid was present in leaves, but not in rhizomes of *P. pseudoaureum*, *P. macrocarpa*, *P colpodes*, *P. plesiororum*, *P. ursipes* Moritz ex C. Chr. and *S. triseriale*. Four species *C. amphostenon* (Kunze ex Klotzsch) Fée, *C. latum*, *P. myriolepis*, and *Polypodium* sp., contain chlorogenic acid in both leaves and rhizomes.

Figure 2D shows the chlorogenic acid content of wild ferns included in this study. Although chlorogenic acid is reported as a phenolic component of *P. aureum* commercial formulations^22^, we did not find a detectable concentration on the wild rhizome, because of its very low concentration (if present). However, five rhizome extracts contain chlorogenic acid: *C. amphostenon, C. latum*, *N. nidulare*, *P. myriolepis,* and *Polypodium* sp., ranging from 0.8 to 4.7 mg/g DM. Figure 2B show chlorogenic acid content in leaves. *P. aureum*’s leaves contain around 3.7 mg/g chlorogenic acid, and another seven ferns contain around 0.8–3.7 mg/g of this metabolite (*S. triseriale*, *Polypodium* sp., *P. plesiosorum*, *P. colpodes*, *P. macrocarpa*, *C. latum*, and *C. amphostenon*). Two fern leaves contain significantly higher chlorogenic acid content (7.9–8.5 g/L): *P. ursipes* and *P. myriolepsis*.

From all these results, we highlight the high quinic acid content in *N. nidulare* and *S. sessilifolium* rhizomes, and *P. macrocapa* leaves. Other elements to consider in the future, to determine the feasibility of industrialization of these wild ferns, are reproduction, culturing, and toxicity. *P. leucotomos* extract is safe for human consumption. Some other ferns are known to contain ptaquiloside (a nor-sesquiterpene), and sesquiterpenes exhibiting mutagenic, teratogenic, clastogenic, and carcinogenic activities^25^.

### Total polyphenolic content of wild ferns

**Figure 3:**
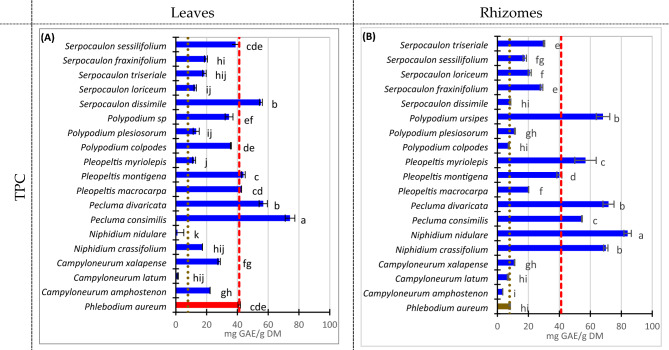
Total polyphenolic content of Costa Rican wild ferns samples (dry base) determined by means of Folin–Ciocalteau method. (**A**) Fern leaves. (**B**) Fern rhizomes. Error bars represent standard deviation. Dashed and dotted lines represent *Phlebodium aureum* leaves and rhizome concentration, respectively. Compact letter display beside the column represents Tuckey’s test. α = 0.05.

Figure 3 summarizes the TPC of the leaves and rhizomes of the ferns. TPC in leaves (Fig. 3A) is found the highest for *P. consimilis* (74.2 mgGAE/gDM). Two other fern leaves contain TPC considerably higher than *P. aureum* leaves (41.2 mgGAE/gDM): *S. dissimile* (55.3 mgGAE/gDM) and *P. divaricata* (56.8 mgGAE/gDM). Other five fern species contain similar TPC (± 6.6 mg mgGAE/gDM) than *P. aureum* leaves: *P. montigena* (43.9 mgGAE/gDM), *P. macrocarpa* (42.64 mgGAE/gDM), *S. sessilifolium* (38.84 mgGAE/gDM), *P. colpodes* (35.8 mgGAE/gDM), and *Polypodium* sp. (34.6 mgGAE/gDM).

Interestingly, most of both leaves’ samples (Fig. 3A) and rhizome’s samples (Fig. 3B) contain higher TPC than *P. aureum* rhizome (commercially used), except *N. nidulare’s* and *C. latum’s* leaves; and, *C. amphostenon*’s, *P. colpodes*’s and *C. latum*’s rhizomes despite the last two are virtually the same TPC.

In rhizomes, seven species have shown the highest TPC particularly high: *N. nidulare* (84.10 mgGAE/gDM), *P. divaricata* (71.73 mgGAE/gDM), *P. ursipes* (68.17 mgGAE/gDM), *N. crassifolium* (70.00 mgGAE/gDM), *P. myriolepis* (56.87 mgGAE/gDM), *P. consimilis* (54.58 mgGAE/gDM), and *P. montigena* (40.00 mgGAE/gDM).

Table 2 compares the highest TPC in our leaves and rhizomes samples with some literature values. There are some ferns such as *Nephrolepis auricata* or *Woodwardia unigemmata* with a considerably higher amount of TPC (318–873 mgGAE/g vs 74–88 mgGAE/g), and also others with lower content such as *Azolla filiculoides* or *Salvinia cucullate*. However, photoprotection or antibacterial activities can be not related in some of them depending on the distribution of individual phenolic compounds and the presence of the bioactive ones.

**Table 2:** TPC in selected ferns extracted with various solvents.

Fern	TPC (mg GAE/g)	Solvent	References
*Nephrolepis auriculata*	590	Methanol	^31^
*Nephrolepis auriculata*	318	Water	^31^
*Matteuccia struthiopteris* (L.) Todar. and *Athyrium multidentatum* (Doll.)	Up to 12	60% ethanol	^32^
*Azolla filiculoides*	27.2	Methanol	^33^
*Woodwardia unigemmata* (Makino)	363	Water	^34^
*Woodwardia unigemmata* (Makino)	873	Methanol	^34^
*Woodwardia unigemmata* (Makino)	31	Hexane	^34^
*Salvinia cucullata*	23.83	Water	^35^
*Salvinia cucullata*	47.16	Ethanol	^35^
*Pecluma consimilis* (leaves)	74.18	75% methanol	This work
*Niphidium nidulare* (rhizome)	84.10	75% methanol	This work

### Antibacterial properties of fern extracts

**Figure 4:**
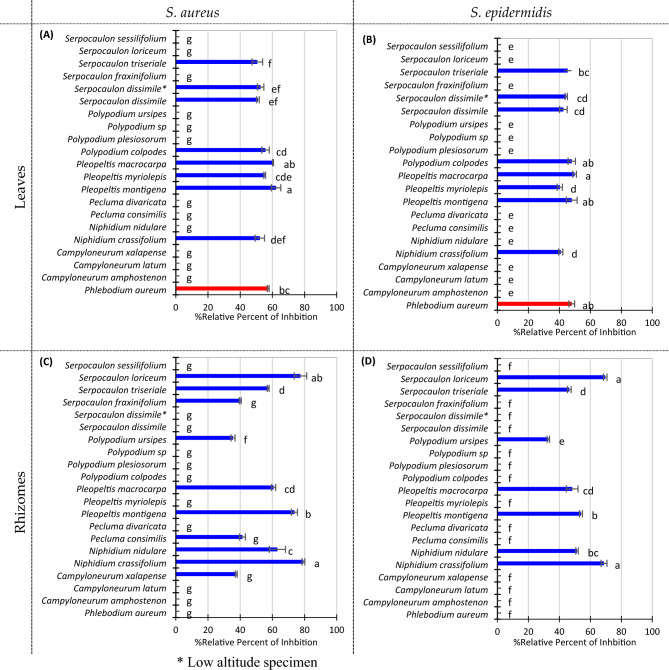
Antibacterial potential of wild fern extracts. (**A**) Leaves extracts against *S. aureus.* (**B**) Leaves extracts against *S. epidermidis.* (**C**) Rhizome extracts against *S. aureus.* (**D**) Rhizome extracts against *S. epidermidis.* 50 μL of 1000 μg/mL of Penicillin/Streptomycin were used as positive control. Error bars represent standard deviation. Compact letter display beside the column represents Tuckey’s test. α = 0.05.

Figure 4 summarizes the relative percent of inhibition of the extracts. The leaves *of N. crassifolium, P. aureum, P. macrocarpa, P. montigena, P. myriolepis, P. colpodes, S. dissimile,* and *S. triseriale* were found active against both *S. aureus* and *S. epidermis*, showing similar relative inhibition between them (around 40–62% respecting the positive control). All the other extracts from leaves do not possess antimicrobial activity.

The activity of rhizome extracts can be divided into three groups. The strongest relative inhibition against *S. aureus* (Fig. 4C) was found in the range of 65–80% for the extracts of *N. crassifolium*, *P. montigena,* and *S. loriceum*. The first two extracts show a similar inhibition against *S. epidermis* (Fig. 4D) too. An intermediate range of the strength of relative inhibition (45–65%) was observed for *N. nidulare*, *P. macrocarpa*, and *S. triseriale,* against both bacteria (*S. aureus* and *S. epidermis*). Extracts from *P. montigena* also showed an intermediate inhibition against *S. epidermis*. Finally, there is a group with a moderate relative inhibition (< 45%), including the rhizomic extracts from *C. xalapense* Fée, *P. consimilis*, and *S. fraxinifolium,* with antimicrobial against *S. aureum*, and the extracts from *P. ursipes* against both *S. aureum* and *S. epidermis*.

Quinic and chlorogenic acids are reported to be antibacterial against several strains of *S. aureus*, *E. coli*, some *bacillus*, and others^26,27^. However, in our results, none of both compounds showed any activity against the strains evaluated here under the same conditions tested for the fern extracts (data not shown). Yet, some other compounds such as flavonoids (flavones, flavonols, flavanones), and organic acids (aliphatic and aromatic acids) are known for being antimicrobial^28^. Therefore, we evaluated the crude extracts of the wild ferns against the gram-negative: *P. aeruginosa* and *E. coli*, and the gram-positive *S. aureus* and *S. epidermidis*. Our results show that the ferns possess antimicrobial against the gram-positive bacteria tested but not against the gram-negative ones (Fig. 4). Remarkably, most skin infections involve gram-positive bacteria, and antibiotic resistance is occurring more frequently in them^29^. Fern extracts are active probably because of synergistic effects between their different compounds.

### Solar protection factor (SPF) of fern extracts

**Figure 5:**
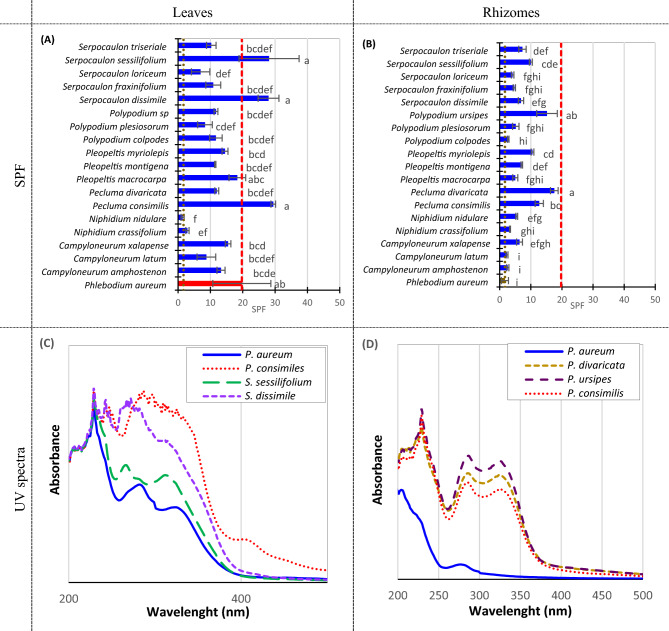
Solar protection factor of Costa Rican wild ferns samples (dry base) at a concentration equivalent to the found in the original dry material, and ultraviolet absorption spectra of selected samples. (**A**) SPF of leaves samples. (**B**) SPF of rhizomes samples. (**C**) UV spectrum of selected leaves samples. (**D**) UV spectrum of selected rhizome samples. For (**A,B**), error bars represent standard deviation, and dashed and dotted lines represent *Phlebodium aureum* leaves and rhizome SPF, respectively. (**C,D**) Spectra were recorded at a constant concentration for all samples. Letters on top of columns represent compact letter display for Tukey’s test (**A,B**). α = 0.05.

Figure 5A,B is showing the SPF for 19 fern species included in this study. Interestingly, almost all the samples have shown higher SPF than *P. aureum* rhizomes, except just *N. nidulare*’s leaves. The highest SPF in leaves (Fig. 5A) is *P. consimiles* (29), *S. sessilifolium* (28), and *S. dissimile* (28). Also, highest SPF in rhizomes is *P. divaricata* (18), *P. ursipes* (15), and *P. consimiles* (13). We did not find a correlationship between TPC, quinic acid, or chlorogenic acid concentration and SPF. UV absorption is probably influenced by more than one compound, and according to Mensul’s equation, compounds with high absorptivity at 300–305 nm are the most important contributors to SPF. It is very likely, most compounds responsible for UV absorption are polyphenols, although, some of them could have maximum absorption at other wavelengths or low molar absorptivities, and this is why there is no correlationship between SPF and TPC.

Selected ultraviolet spectra containing *P. aureum* and the samples with the highest SPF are shown in Fig. 5C,D. All the samples cover the whole regions of UVA (315–399 nm), UVB (280–314 nm), and UVC (100–279). However, *P. consimilis* and *P. dissimile’s* leaves spectra have wider absorption peaks at the UVA region than *P. aureum* and *S. sessilifolium*. In the rhizomes (Fig. 5D), *P. divaricata*, *P. ursipes*, and *P. consimilis* show a more intense absorption in the whole spectrum, but the effect is also more notorious in the UVA region.

### Acclimatization effect on metabolite production

Four out of the six ferns selected for acclimatization were used to determine metabolite concentrations: *C. latum*, *N. nidulare*, *P. aureum*, and *S. attenuatum*. We considered various characteristics to choose them, such as quinic and chlorogenic acids production of wild types, feasibility for pot cultivation (long-creeping rhizome species such as *P. macrocarpa* and *P. loriceum* need some characteristics from the wild and cannot grow in a standardized substratum), sample preservation and their availability (*N. crassifolium* is a casual specie, and a population of this fern cannot be found). *S. attenuatum* has not been included in the initial screening, although it is close to other *Serpocaulon*.

Table 3 shows metabolite content for greenhouse-grown ferns. The production of metabolites differs significantly from wild ones (Fig. 2). Most acclimatized ferns increase quinic acid concentration, respecting their wild specimens. QA content increases from 0 to 3.64 (leaves), 2.29 (rhizomes) mg/g for *C. latum* to 13.94 mg/g for *N. nidulare* leaves, and 8.31 mg/g for *P. aureum* leaves. Rhizome QA content increases by 18% (13.31 the best acclimatized vs 11.29 mg/g) for *P. aureum* and 5% (17.97 vs 17.03 mg/g) for *N. nidulare.* CGA concentration is very similar for wild and acclimatized fern specimens (± 2 mg/g). The most important change is observed for *C. latum* leaves (2.42 mg/g in wild vs 9.85 mg/g acclimatized).

**Table 3:** Metabolite concentration and solar protection factor of acclimatized fern samples at different conditions based on dry mass (± standard deviations).

Sample	Shade (%)	QA (mg/g DM)	CGA (mg/g DM)	TPC (mg GAE/g DM)	SPF
*C. latum* leaves	50	ND	ND	ND	ND
	70	3.64 ± 0.08	9.85 ± 0.20	17.61 ± 0.13	ND
*C. latum* rhizomes	50	ND	ND	ND	ND
	70	2.19 ± 0.05	0.21 ± 0.00	ND	ND
*N. nidulare* leaves	50	13.94 ± 0.20	1.39 ± 0.01	24.97 ± 0.94	ND
	70	10.90 ± 0.30	1.70 ± 0.10	ND	ND
*N. nidulare* rhizomes	50	17.97 ± 0.43	0.42 ± 0.01	26.72 ± 1.29	3.42 ± 0.23
	70	13.92 ± 0.98	1.85 ± 0.10	ND	ND
*P. aureum* leaves	50	8.31 ± 0.88^a^	3.62 ± 0.19^a^	38.35 ± 0.24^a^	ND
				47.59 ± 3.26^b^	
	70	8.29 ± 0.34^a^	0.99 ± 0.02^a^	ND	ND
*P. aureum* rhizomes	50	13.31 ± 0.57^a^	0.18 ± 0.00^a^	24.69 ± 1.55^a^	4.61 ± 1.39^a^
				8.79 ± 0.40^b^	3.87 ± 2.98^b^
	70	4.38 ± 0.35^a^	0	ND	ND
*S. attenuatum* leaves	50	ND	ND	ND	ND
	70	14.77 ± 1.46	2.50 ± 0.11	ND	ND
*S. attenuatum* rhizomes	50	ND	ND	ND	ND
	70	13.83 ± 0.61	1.54 ± 0.03	22.40 ± 0.65	4.42 ± 0.18

*QA* quinic acid, *CGA* chlorogenic acid, *TPC* total polyphenolic content, *SPF* solar protection factor, *DM* dry mater, *ND* non determined.

^a^High-altitude specimen.

^b^Low-altitude specimen.

TPC is increased with acclimatization for most ferns: *C. latum* increases from 1.28 to 17.61 mgGAE/gDM (leaves) and 1.04 to 24.97 (mgGAE/gDM), *P. aureum* from 41.18 to 47.59 mgGAE/gDM (leaves) and 7.89 to 24.69 mgGAE/gDM, and *N. nidulare*’s leaves from 1.04 to 24.97 mgGAE/gDM (respecting best acclimatization condition). Only *N. nidulare*’s rhizomes decrease TPC from 84.01 to 26.72 mgGAE/gDM.

According to the literature, phenolic compounds can exhibit a UV-protectant mechanism, the same responsible for human skin protection. Then, higher altitudes and less shade can induce the production due to the higher exposition to UV sunlight^30^. Then, QA and TPC decrease with the increase in the shade, for all samples where 50 and 70% shade were recorded. On the other hand, many wild specimens showing high values of those compounds live on top of trees and places off the shade. Also, the SPF of *N. nidulare’s* rhizome extract decreases from 5.47 to 3.42 with acclimatization and increases from 1.69 to 4.61 for *P. aureum*’s rhizomes, although those differences are not significant.

## Conclusions

We demonstrate wild ferns have an interesting potential for skin healing formulations. 19 out of 20 species included in this study had shown either some content of quinic and/or chlorogenic acid and/or antimicrobial activity. Several ferns contain higher concentration of QA and CGA than ferns used commercially. Also 17 out of 19 species showed TPC higher than *P. aureum* rhizomes. Most wild ferns have higher SPF than *P. aureum* rhizomes, demonstrating their UV protectant capabilities*.* Ten out of twenty evaluated ferns demonstrated antimicrobial properties against *S. aureus* and *S. epidermis*. We demonstrated that fern’s acclimatization is feasible and increases QA content and TPC in most cases. Although we did not find a linear co-relationship between metabolites tested and UV protectant properties, this last effect is probably due to synergistic effects between some specific polyphenols and/or other polyunsaturated molecules. Toxicity (for oral formulations), cytotoxicity, and stability of the extracts needs to be studied in future, however, our findings reveals a very interesting candidates for skin products.

### Supplementary Information

Supplementary Information.

## Data Availability

Data will be available upon request. If data or materials are needed, please contact Victor Álvarez-Valverde, email: victor.alvarez.valverde@una.cr.
