# Supplementary material for: Exploration of photoprotective and antibiotic activity of wild Polypodiaceae ferns from Costa Rica
Source: Sci Rep. 2024 Jan 18;14:1602. doi: 10.1038/s41598-023-50281-3 (PMC10796928; doi:10.1038/s41598-023-50281-3)
Supplement: Supplementary file 1 — Supplementary Information. [file 41598_2023_50281_MOESM1_ESM.docx]

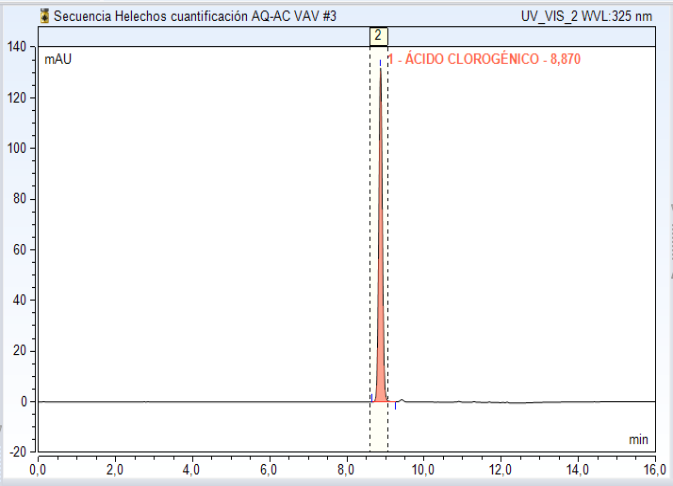


Figure S1. Chlorogenic acid chromatograph at 325 nm. Selected signal correspond to chlorogenic acid with a retention time of 8.87 min.


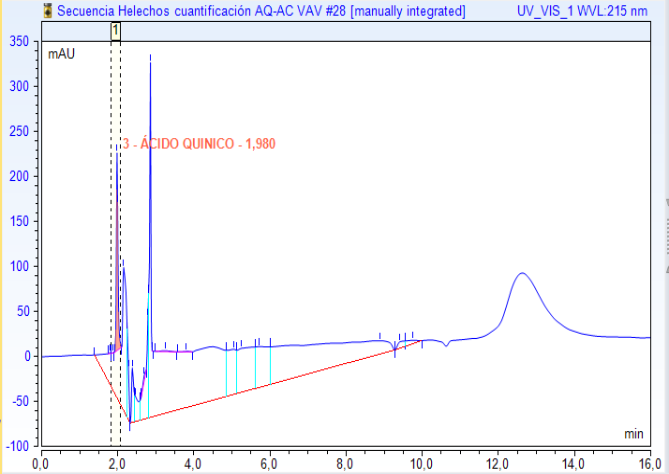


Figure S2. Quinic acid chromatograph at 215 nm. Selected signal correspond to quinic acid with a retention time of 1.98 min.

|  |
| --- |
|  |

Figure S3. Optimization of the extraction procedure. Error bars represent standard deviation (I) Solvent selection (A) methanol 75%, (B) methanol 100%, (C) ethanol, (D) methanol:ethanol (1:1). (II) Number of extraction cycles.

Supplementary procedures

Solvent selection

We tested 4 solvent as extractants: (A) 75% methanol, (B) methanol, (C) ethanol, and (D) ethanol:methanol (1:1). Approximately 0.1000 g of the composite samples of leaves and rhizomes were measured in test tubes. Extractions were carried out as follows: 3.0 mL of solvent were added to each sample, and they were mixed for 30 seconds using a vortex mixer. Then, the test tubes were placed in an ultrasonic bath for 10 minutes at room temperature and subsequently centrifuged for 8 minutes at 3200 rpm. The supernatant liquid from each sample was removed with a Pasteur pipette and added to the respective 10.00 mL volumetric flask. Three extractions were performed to each sample, combined and the volumes of each flask were completed with the respective solvent.

A UV-Vis spectrophotometer, PG Instruments Limited, model T80+, was used to evaluate the response of each sample extract in the different solvents and select the one that showed the highest A/m ratio. Scans were performed in the range of (200 to 400) nm to define a wavelength (λ) that would allow for the comparison of the extraction capacity of the different solvents. After choosing λ, the absorbance of all the extracted samples was determined, diluting 150.00 µL of the extract in 3.0 mL of 95% ethanol in the cuvette before each reading. This was done to prevent the readings from exceeding the linear range of the detector's response. The readings were accompanied by 95% ethanol as a blank.

Evaluation of the number of extractions

Once the solvent was chosen, the number of extractions needed to obtain the highest yield was determined. The mass of 0.1000 g of each composite sample was measured in a test tube in triplicate for each extraction test, ranging from one to four extractions of 2.00 mL of solvent each. These were added to 10.00 mL volumetric flasks, brought to volume, and the absorbances at the previously established λ were read. The A/m ratio was used as a criterion to select the optimal number of extractions.

Results for solvent selection and number of extractions is presented in supplementary figures S3 (A) and (B), respectively
